# LINC00659 Inhibits Hepatocellular Carcinoma Malignant Progression by Blocking Aerobic Glycolysis through FUS Recruitment and SLC10A1 Modulation

**DOI:** 10.1155/2023/5852963

**Published:** 2023-05-17

**Authors:** Bin Chen, Xin Xu, Wei Wu, Ke Zheng, Yijun Yu

**Affiliations:** Department of Hepatobiliary Surgery, The Affiliated Hospital of Hangzhou Normal University, Zhejiang, Hangzhou 310011, China

## Abstract

Hepatocellular carcinoma (HCC) is a malignant type of liver cancer that poses severe threat to human health worldwide. Aerobic glycolysis is a hallmark of HCC and facilitates its progression. Solute carrier family 10 member 1 (SLC10A1) and long intergenic non-protein coding RNA 659 (LINC00659) were detected to be downregulated in HCC cells, yet their potential functions underlying HCC progression remained unidentified. In the current work, colony formation and transwell assays were used to detect HCC cells (HepG2 and HuH-7) proliferation and migration *in vitro* study. The quantitative real-time polymerase chain reaction (qRT-PCR) and western blot assays were used for gene/protein expression determination. Seahorse assay was performed for aerobic glycolysis assessment. RNA immunoprecipitation (RIP) and RNA pull-down assays were conducted for detection of the molecular interaction between LINC00659 and SLC10A1. The results showed that overexpressed SLC10A1 significantly suppressed the proliferation, migration, and aerobic glycolysis in HCC cells. Mechanical experiments further demonstrated that LINC00659 positively regulated SLC10A1 expression in HCC cells by recruiting fused protein in sarcoma (FUS). Our work elucidated that LINC00659 inhibited HCC progression and aerobic glycolysis via the FUS/SLC10A1 axis, revealing a novel lncRNA–RNA-binding protein–mRNA network in HCC, which might provide potential therapeutic targets for HCC.

## 1. Introduction

Hepatocellular carcinoma (HCC) is one of the most common and malignant type of liver cancer, with its death rate ranking the fourth in cancer-related death records worldwide [1]. It has been found that environmental changes, along with epigenetic alterations, may result in HCC progression [2]. The clinical behavior of HCC can grow gradually from asymptomatic to life-threatening, including acute complications. Many HCC patients, especially diagnosed at an early stage, have no obvious clinical symptoms related to tumor [3]. Moreover, HCC has been found to cause cirrhosis, which is probably induced by reduced growth capacity of hepatocytes [4]. The diagnosis of HCC requires more than one imaging modality and biomarkers, much delaying the detection and worsening the prognosis for terminal-staged tumors [5]. Although surgical removal is the best prognostic treatment for tumor, the diagnosis at an early stage is of great importance [6], to improve the prognosis and the survival significantly.

Aerobic glycolysis was first found in HCC and acts as a marker of liver cancer. It participates in immune evasion, proliferation, metastasis, angiogenesis, invasion, and drug resistance in HCC [7]. Glucose transporter protein type 1 (GLUT1) and the three rate-limiting enzymes in the glycolytic process, including hexokinase 2 (HK2), lactate dehydrogenase (LDHA), and phosphoglycerate kinase 1 (PGK1), play important roles in aerobic glycolysis of HCC and can be regulated by many mechanisms, such as the signaling pathways and long non-coding RNAs (lncRNAs) [8–14]. Therefore, it is necessary to explore the regulators of aerobic glycolysis in HCC.

As important regulatory genes [15], lncRNAs have been found to exert crucial effects on regulating a variety of physiological activities of cancers, especially in HCC [16]. For example, upregulation of SNHG15 associates closely with HCC development [17]. GABPB1-AS1 is involved in controlling oxidative stress during erastin-induced ferroptosis in HCC cells [18]. Additionally, Yang et al. have stated that SNHG1 and SNHG3 promote the proliferative, migratory, and invasive abilities of HCC cells [19]. However, there are few studies analyzing the functions of long intergenic non-protein coding RNA 659 (LINC00659) in malignant diseases [20, 21], HCC included.

Solute carrier family 10 member 1 (SLC10A1), highly expressed in the liver, encodes the Na^+^-taurocholate co-transporting polypeptide [22–24] and has been proven to inhibit HCC cell glycolysis by glucose utilization, lactate production, and extracellular acidification ratio [25]. Studies have revealed that the expression levels of SLC10A1 in tumors are consistently lower than that in normal tissue. Downregulation of SLC10A1 is correlated with poor survival outcome and recurrence-free survival in patients with HCC [26], but whether SLC10A1 has interaction with other regulatory factors in HCC remains unknown. Additionally, fused protein in sarcoma (FUS), a famous RNA-binding protein (RBP), has been found to be significantly downregulated in HCC tissues and decreases cell viability, migration, and invasion with its overexpression [27]. Of note, a previous report has indicated that lncRNA LINC01419 interacts with FUS to stabilize specific mRNA, therefore facilitating HCC growth [28].

According to the mentioned above, we proposed the hypothesis that LINC00659 might stabilize SLC10A1 mRNA to affect HCC progression and aerobic glycolysis through FUS recruitment. Our results elucidated an original lncRNA–RBP–mRNA regulatory network, which offered the potential therapeutic targets for HCC.

## 2. Materials and Methods

### 2.1. Clinical Samples

The tumor and adjacent noncancerous specimens were surgically dissected from 46 patients with HCC at the Affiliated Hospital of Hangzhou Normal University. No chemotherapy or radiotherapy had been performed on these patients before surgery. Cancer and noncancerous regions were checked by two pathologists who were not directly involved in this study. All enrolled patients were informed of the aim of this study. The experimental procedures were authorized by the Affiliated Hospital of Hangzhou Normal University Institutional Ethics Committee. The experiments were performed following the Declaration of Helsinki.

### 2.2. Cell Culture

Human normal hepatocytes (THLE-2) were purchased from ATCC (CRL-2706, Manassas, VA, USA), and human HCC cell lines (HepG2, HuH-7, and Li-7) were acquired from Procell (CL-0103; CL-0120; CL-0139, Wuhan, Hubei, China). DMEM (CM-0120, Procell, Wuhan, Hubei, China) containing 10% fetal bovine serum (FBS) and 1% penicillin/streptomycin was used for HepG2, HuH-7, and Li-7 cells. BEGM (CC3170, Lonza/Clonetics Corporation, Walkersville, MD) containing 5 ng/mL epidermal growth factor (EGF), 70 ng/mL phosphoethanolamine, and 10% FBS was used for THLE-2 cells. All cells were cultured in 5% CO_2_ at 37°C.

### 2.3. Cell Transfection

Transfection vectors, pcDNAs (pcDNA-3.1/pcDNA-3.1-SLC10A1 and pcDNA-3.1/pcDNA-3.1-LINC00659) and shRNAs (sh-NC/sh-FUS-1/2 and sh-NC/sh-SLC10A1-1/2), were designed and synthesized from Genechem (Shanghai, China). Vectors were transfected into HepG2 and HuH-7 cells using Lipofectamine 2000 (Invitrogen, Grand Island, NY) for 24 hours according to manufacturer's instructions.

### 2.4. Colony Formation Assay

HepG2 and HuH-7 cells were seeded into 6-well plates (1 × 10^3^ cells/well) and routinely cultured for 14 days [29]. Colonies were fixed with 4% paraformaldehyde for 30 minutes, stained with 1% crystal violet for 20 minutes (500 *μ*L/well, Beyotime, Shanghai, China), washed several times, dried, and photographed (Nikon ECLIPSE Ti, Japan). The colonies larger than 50 cells were counted.

### 2.5. Cell Counting Kit-8 (CCK-8) Assay

HepG2 and HuH-7 cells were seeded into 6-well plates (1 × 10^3^ cells/well), and incubated overnight, and 10 *μ*L CCK-8 solution (CK04-11, Dojindo, Kumamoto, Japan) was added. After 2 hours of incubation, absorbance at 450 nm was measured using a microplate reader (spectramax plus384, Molecular Devices, USA).

### 2.6. Transwell Assay

An 8 *μ*m-pore polycarbonate membrane Boyden chamber was used to detect cell migration (Corning, USA). The upper chamber was coated with 2 × 10^5^ HepG2 or HuH-7 cells in serum-free medium, while the bottom chamber was filled with 0.5 mL of complete medium with 10% FBS. Then the chamber was incubated in 5% CO_2_ for 1 day at 37°C. Subsequently, the cells on the upper chamber were removed, while those invading onto the lower chamber were fixed with methanol. Crystal violet (2%) was used for staining for 10 minutes. For each membrane, at least five random visual fields were selected to count the invaded cells under a microscope (BHNK-PH001, Nikon Corporation; magnification, ×100).

### 2.7. Wound-Healing Assay

The cells were scratched with a sterile pipette tip when cultured to 100% confluent monolayer. Plates were rinsed with PBS twice to remove detached cells, replaced with fresh serum-free medium, and placed in an incubator with 5% CO_2_ at 37°C. Cells were removed at 0 and 24 hours, with images photographed under an inverted microscope (Olympus, Japan). Wound closure = (initial width − width at 24 hours)/initial width × 100%.

### 2.8. Seahorse Assay

Seahorse Bioscience Extracellular Flux Analyzer (XFe24; Seahorse Bioscience) was utilized to survey the extracellular acidification rate (ECAR) and oxygen consumption rate (OCR) of HepG2 or HuH-7 cells. Briefly, transfected HCC cells (1 × 10^4^) were seeded into the 24-well cell culture plate (Seahorse Bioscience). The hydrated XF24 sensor cartridge was loaded with oligomycin (1 *μ*M final concentration) and protonophore trifluoromethoxy carbonyl cyanide phenylhydrazone (FCCP, 1 *μ*M final concentration). The oxygen consumption rate and extracellular acidification rate were measured in accordance with the manufacturer's instructions and expressed in mpH/min and pmoles/min, respectively.

### 2.9. RIP

Magna RNA immunoprecipitation (RIP) RBP Immunoprecipitation Kit (17-700, Sigma-Aldrich, USA) was used for RIP assay following manufacturer's protocol. Antibodies against FUS (ab23439, 1/1000, Abcam, Cambridge, UK) and IgG (ab182931, 1000, Abcam, Cambridge, UK) were used for positive and negative controls. The quantitative real-time polymerase chain reaction (qRT-PCR) was used for detecting the enrichment of LINC00659 or SLC10A1 3′UTR, respectively. Then, the binding complex was collected for agarose gel electrophoresis, and the existence of LINC00659 and SLC10A1 was directly observed.

### 2.10. RNA Pull-Down Assay

LINC00659 or SLC10A1 was transcribed using T7 RNA polymerase, purified using RNeasy Plus Mini Kit, and labeled with Biotin RNA Labeling Mix. Biotinylated LINC00659 sense or LINC00659 anti-sense, which were all synthesized by GeneChem, were added to proteins extracted from cells and co-incubated with streptavidin agarose beads. Gel electrophoresis was used to separate FUS. Mass spectrometry was performed to analyze eluted lncRNA-binding proteins. PEAKS Studio 8.5 was used for protein identification and quantification. At last, western blotting was used to analyze the eluted proteins.

### 2.11. RNA Stability Assay

After transfection, HepG2 or HuH-7 cells were treated with 5 mg/mL actinomycin D (D23070, Acmec, Shanghai) for 0, 6, 12, 18, and 24 hours at 37°C. Total RNA was isolated using an RNeasy Kit (74104, Qiagen) under conditions recommended by the manufacturer. The mRNA levels of SLC10A1 following actinomycin treatment were subsequently measured using RT-qPCR.

### 2.12. Quantitative Real-Time Polymerase Chain Reaction

Total RNA was isolated from HepG2 or HuH-7 cells using the Trizol reagent (Invitrogen, USA) strictly following the manufacturer's protocol. The RNA was reverse-transcribed into cDNA using a reverse transcription reagent kit (RP1105, Solarbio, Beijing, China). The PCR was performed using SYBR® Green Master Mix (Takara, Japan) and Light Cycler 480 II System (Roche, China). The reaction conditions were as follows: initial denaturation at 95°C for 10 minutes, followed by 40 cycles of 95°C for 10 seconds, 60°C for 34 seconds. The relative expression was calculated using 2^−*ΔΔ*Ct^ method. Primer sequences were as follows: LINC00659: F: 5′-ATGCTTAACAGGAGGCTCC-3′, R: 5′-ATCCTTTCAGGAGGGAGGT-3′; SLC10A1: F: 5′-AACCTCAGCATTGTGATGAC-3′, R: 5′-CCTGGAGTAGATGTACAGGA-3′; FUS: F: 5′-GAGGATTTCCCAGTGGAGG-3′, R: 5′-TCTCACAGGTGGGATTAGGA-3′; *β*-actin: F: 5′-ACTCTTCCAGCCTTCCTTCC-3′, R: 5′-CGTCATACTCCTGCTTGCTG-3′.

### 2.13. Western Blot

The protein extraction protocol from cancer cells was previously reported [30]. Briefly, cells were collected and lysed using Radio Immunoprecipitation Assay (RIPA) protein extraction reagent (Beyotime) with a protease inhibitor cocktail (Roche, IN, USA). Equal amounts of protein lysate were quantified using BCA kit (Thermo Fisher, USA), separated by 10% sodium dodecyl sulfate–polyacrylamide gel (SDS–PAGE) electrophoresis, and transferred onto a polyvinylidene fluoride (PVDF) membrane (Millipore, Billerica, MA, USA). The membrane was then sealed with 5% non-fat milk for 1 hour, and incubated with primary antibodies (Abcam, Cambridge, UK) against: FUS (ab84078, 1/1000), SLC10A1 (ab131084, 1/1000), *β*-actin (ab8227, 1/1000), GLUT1 (ab14683, 1/2500), HK2 (ab227198, 1/5000), LDHA (ab47010, 1/1000), PGK1 (ab154613, 1/1000) overnight at 4°C, with GAPDH (ab9485, 1/2500) as control. After washing, the membrane was incubated with goat anti-rabbit secondary antibody (ab96899, 1/1000, Abcam, Cambridge, UK) at 37°C for 1 hour. The protein bands were finally detected using enhanced chemiluminescence reagents (Bio-Rad, Hercules, CA, USA) according to manufacturer's instructions.

### 2.14. Immunohistochemistry

Sample blocks were first sectioned at 4 *μ*m thickness, deparaffinized in xylene, and rehydrated in ethanol and deionized water. After heat-mediated antigen retrieval, slides were stained with anti-FUS antibody and anti-SLC10A1 overnight at 4°C. The next day, the slides were washed with 1 × TBS-T and stained with Horseradish Peroxidase (HRP)-conjugated goat antirabbit IgG secondary antibody for 1 hour. After counterstaining using hematoxylin for 30 seconds, the slides were washed and stained with 3,3′-diaminobenzidine. The results were examined and photographed under a light microscope (Leica Microsystems, USA).

## 3. Results

### 3.1. SLC10A1 Expressed Lowly in HCC Cells and Suppressed Cell Proliferation and Migration

**Figure 1:**
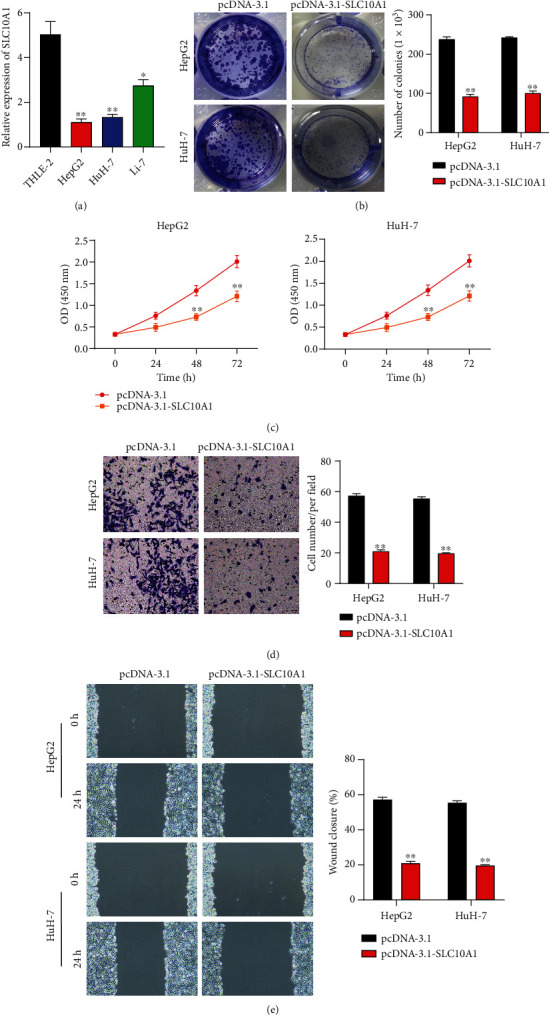
SLC10A1 expressed lowly in HCC cells and suppressed its proliferation and migration. (a) The expression of SLC10A1 in human normal hepatocytes (THLE-2) and human hepatocellular carcinoma cell lines (HepG2, HuH-7, and Li-7) was determined by qRT-PCR. (b) The proliferation of HepG2 and HuH-7 cells with SLC10A1 overexpression was detected by colony formation assay. (c) The proliferation of HepG2 and HuH-7 cells with SLC10A1 overexpression was detected by CCK-8 assay. (d) The migration of HepG2 and HuH-7 cells with SLC10A1 overexpression was detected by transwell assay. (e) The migration of HepG2 and HuH-7 cells with SLC10A1 overexpression was detected by wound-healing assay. ∗*P* < 0.05, ∗∗*P* < 0.01.

Statistics from Gene Expression Profiling Interactive Analysis (GEPIA) (http://gepia.cancer-pku.cn/) showed that the SLC10A1 expression level is decreased in HCC tissues and is associated with poor patient prognosis (Figures S1(a) and S1(b)). In clinical samples, protein expression of SLC10A1 through immunohistochemistry differed between HCC tissues and normal tissues, and the expression level of SLC10A1 was higher in normal tissues than in HCC tissue (Figure S1(c)). Simultaneously, SLC10A1 was lowly expressed in human hepatocyte carcinoma cells (HepG2, HuH-7, and Li-7), with the lowest in HepG2 and HuH-7 cells, as detected by qRT-PCR (Figure 1(a)). Thus both HepG2 and HuH-7 cells were chosen for the follow-up experiments. To identify the role of SLC10A1 in HCC, both selected cells were transfected with pcDNA-3.1-SLC10A1, and transfection efficiency was verified by qRT-PCR (Figure S1(d)). As evidenced in colony formation assay and CCK-8 staining, increased SLC10A1 expression suppressed cell proliferation (Figures 1(b) and 1(c)). Similarly, transwell and wound-healing assays also uncovered that SLC10A1 overexpression hindered cell migration (Figures 1(d) and 1(e)). These results suggested that SLC10A1 had significantly low expression in HCC cells and suppressed cell proliferation and migration.

### 3.2. SLC10A1 Suppressed Aerobic Glycolysis in HCC Cells

**Figure 2:**
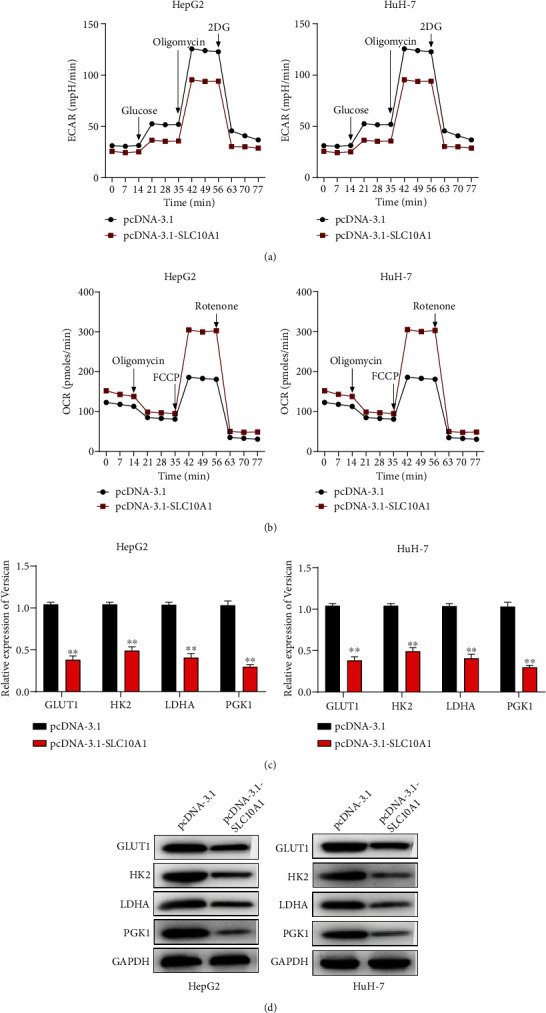
SLC10A1 suppressed aerobic glycolysis in HCC cells. (a) The ECARs of HepG2 and HuH-7 cells with SLC10A1 overexpression were assessed. (b) The OCRs of HepG2 and HuH-7 cells with SLC10A1 overexpression were assessed. (c) The mRNA expression of GLUT1, HK2, LDHA, and PGK1 in HepG2 and HuH-7 cells transfected with pcDNA-3.1-SLC10A1 was determined by qRT-PCR. (d) The protein expression of GLUT1, HK2, LDHA, and PGK1 in HepG2 and HuH-7 cells transfected with pcDNA-SLC10A1 was determined by western blot. ∗∗*P* < 0.01.

It is well-known that aerobic glycolysis plays an essential part for HCC cell proliferation and migration [31]. Therefore, we hypothesized whether SLC10A1 had potential in modulating the processing of aerobic glycolysis. Seahorse assay showed that in HepG2 and HuH-7 cells, overexpressed SLC10A1 suppressed ECAR, indicating decreased glycolysis flux and glycolytic capacity (Figure 2(a)). On the contrary, enhanced SLC10A1 expression promoted OCR, indicating increased basal respiration and adenosine triphosphate (ATP) production (Figure 2(b)). Moreover, the impacts of SLC10A1 overexpression on the mRNA and protein levels of glucose metabolism-related genes (GLUT1, HK2, LDHA, and PGK1) were assessed. The outcome displayed that SLC10A1 overexpression reduced the mRNA and protein levels of these genes, which were detected by qRT-PCR and western blot analyses (Figures 2(c) and 2(d)). These results suggested that SLC10A1 suppressed the aerobic glycolysis in HCC cells.

### 3.3. LINC00659 Positively Regulated SLC10A1 Expression in HCC Cells

**Figure 3:**
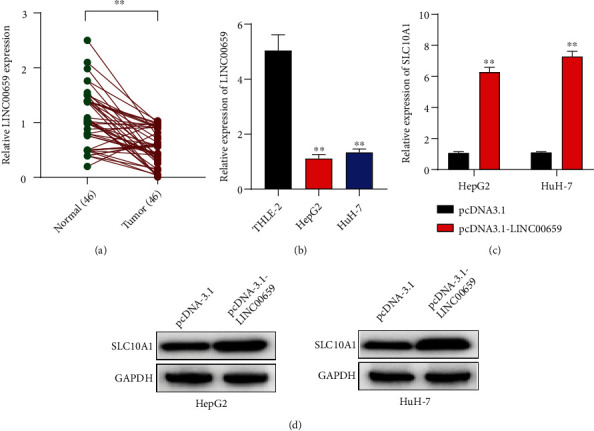
LINC00659 positively regulated SLC10A1 expression in HCC cells. (a) The expression level of LINC00659 in 46 pairs of HCC tissues and adjacent noncancerous specimens was calculated using qRT-PCR. (b) The expression of LINC00659 in human normal liver cell lines (THLE-2) and human hepatocyte carcinoma cell lines (HepG2 and HuH-7) was determined by qRT-PCR. (c) The expression of SLC10A1 in HepG2 and HuH-7 cells transfected with pcDNA3.1-LINC00659 was measured by qRT-PCR. (d) The expression of SLC10A1 in HepG2 and HuH-7 cells transfected with pcDNA3.1-LINC00659 was measured by western blot. ∗∗*P* < 0.01.

We further explored the upstream molecular mechanism of SLC10A1 in HCC. We screened lncRNA with significantly low expression in HCC tissues and then analyzed using GSE101728 database with the filter criteria of *P* value <0.5 and logFC < −3. LINC00659 was found to be obviously downregulated in HCC tissues and had not been specifically reported in HCC. More importantly, GEPIA database also presented the low expression of LINC00659 in HCC tissues (Figure S2(a)), and its expression was positively correlated with SLC10A1 expression in HCC (Figure S2(b)). To validate the downregulation of LINC00659 in HCC, we determined the LINC00659 levels in 46 clinical samples with HCC and paired adjacent noncancerous specimens using qRT-PCR analyses. LINC00659 expression levels were lower in HCC tissues than in the adjacent noncancerous specimens (Figure 3(a)). Therefore, LINC00659 was selected as the study subject. LINC00659 had significantly low expression in HepG2 and HuH-7 cells, as detected by qRT-PCR (Figure 3(b)). Besides, we increased LINC00659 expression in HepG2 and HuH-7 cells (Figure S2(c)) and found that the expression of SLC10A1 at mRNA and protein levels was remarkably promoted in HepG2 and HuH-7 cells transfected with pcDNA3.1-LINC00659 (Figures 3(c) and 3(d)). These results suggested that LINC00659 had low expression and positively regulated SLC10A1 expression in HCC cells.

### 3.4. LINC00659 Stabilized SLC10A1 mRNA by Recruiting FUS

**Figure 4:**
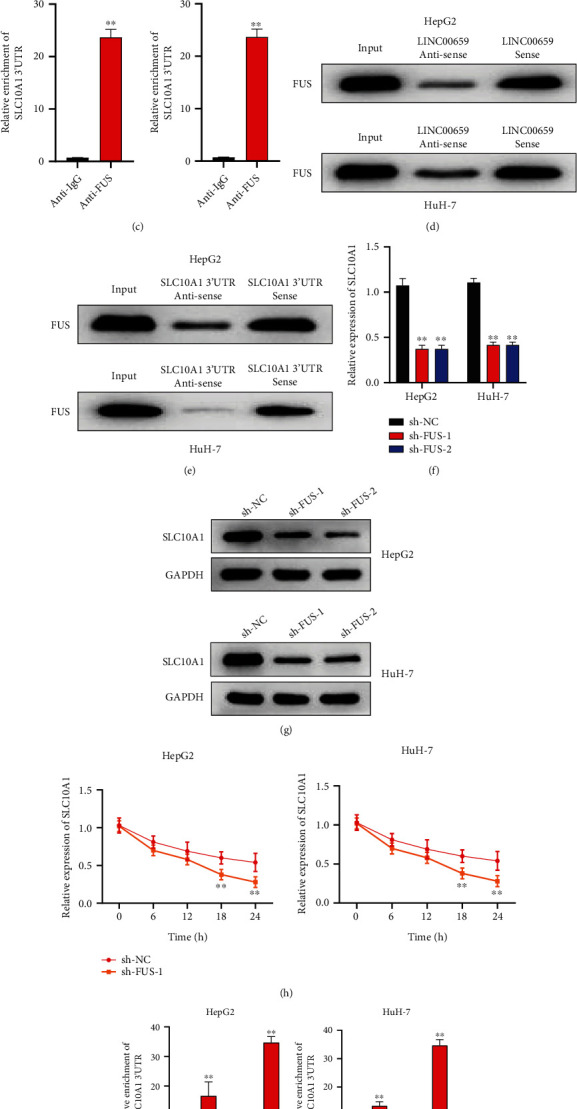
LINC00659 stabilized SLC10A1 mRNA by recruiting FUS. (a) The Venn diagram of LINC00659 RBP and SLC10A1 RBP. (b) The enrichment of LINC00659 in HepG2 and HuH-7 cells was determined by RIP assay. (c) The enrichment of SLC10A1 3′UTR in HepG2 and HuH-7 cells was determined by RIP assay. (d) The interaction of FUS and LINC00659 in HepG2 and HuH-7 cells was assessed by RNA pull-down assay. (e) The interaction of FUS and SLC10A1 3′UTR in HepG2 and HuH-7 cells was assessed by RNA pull-down assay. (f) The expression of SLC10A1 in HepG2 and HuH-7 cells transfected with sh-FUS was examined by qRT-PCR. (g) The protein level of SLC10A1 in HepG2 and HuH-7 cells transfected with sh-FUS was examined by western blot. (h) The stability of SLC10A1 mRNA was detected by qRT-PCR in HepG2 and HuH-7 cells transfected with sh-FUS after actinomycin D treatment. (i) Relative enrichment of SLC10A1 3′UTR in HepG2 and HuH-7 cells transfected with pcDNA3.1-LINC00659/pcDNA3.1 was assessed by RIP assay. ∗∗*P* < 0.01.

The potential mechanism of LINC00659 regulating SLC10A1 expression continued to probe. Through starBase (https://starbase.sysu.edu.cn/) website, we predicted respective RBPs of LINC00659 and SLC10A1 (Figure S3(a)) and discovered that only FUS could bind to both LINC00659 and SLC10A1, as shown in Venn diagram (Figure 4(a)). RIP experiments also suggested that both LINC00659 and SLC10A1 were preferentially enriched in FUS precipitates, confirming the above prediction (Figures 4(b) and 4(c)). Meanwhile, RNA pull-down results further proved that LINC00659 and SLC10A1 could combine with FUS, respectively (Figures 4(d) and 4(e)). In clinical samples, protein expression of FUS through immunohistochemistry differed between HCC tissues and normal tissues, and the expression level of FUS was higher in normal tissues than in HCC tissue (Figure S3(b)). We next knocked down FUS expression in HepG2 and HuH-7 cells (Figure S3(c)) and observed that the mRNA and protein levels of SLC10A1 were markedly declined upon FUS silence (Figures 4(f) and 4(g)). For all we know, FUS acts as a member of RBPs, which can be involved in the modulation of mRNA stability [32]. Herein, we treated HepG2 and HuH-7 cells with actinomycin D. Notably, qRT-PCR analysis indicated that the half-life of SLC10A1 mRNA was distinctly lessened upon FUS deficiency (Figure 4(h)). In addition, it was shown that compared to the pcDNA-3.1 group, the affinity of SLC10A1 3′UTR and FUS in the pcDNA-3.1-LINC00659 group was higher (Figure 4(i)), suggesting that LINC00659 stabilized SLC10A1 mRNA via recruiting FUS. These results demonstrated that LINC00659 stabilized SLC10A1 mRNA and promoted its expression by recruiting FUS.

### 3.5. LINC00659 Suppressed HCC Progression and Aerobic Glycolysis by Regulating SLC10A1

**Figure 5:**
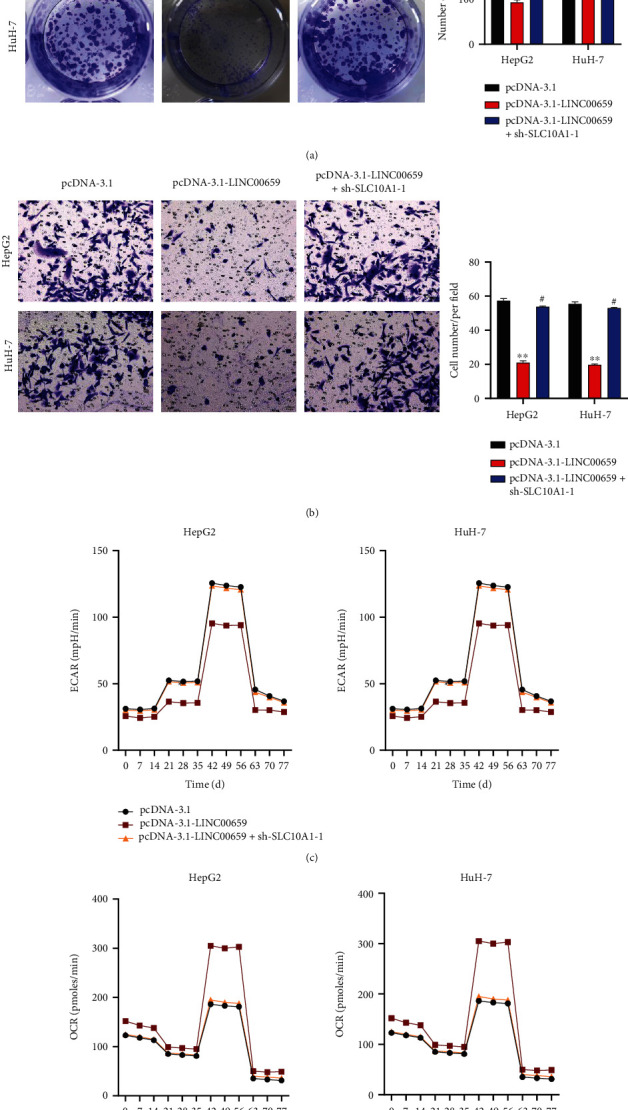
LINC00659 suppressed HCC progression by regulating SLC10A1. (a) Colony formation analysis of HepG2 and HuH-7 cells transfected with pcDNA3.1/pcDNA3.1-LINC00659/pcDNA3.1-LINC00659 + sh-SLC10A1-1. (b) Transwell analysis of HepG2 and HuH-7 cells transfected with pcDNA3.1/pcDNA3.1-LINC00659/pcDNA3.1-LINC00659 + sh-SLC10A1-1. (c) ECARs of HepG2 and HuH-7 cells transfected with pcDNA3.1/pcDNA3.1-LINC00659/pcDNA3.1-LINC00659 + sh-SLC10A1-1. (d) OCRs of HepG2 and HuH-7 cells transfected with pcDNA3.1/pcDNA3.1-LINC00659/pcDNA3.1-LINC00659 + sh-SLC10A1-1. ∗∗*P* < 0.01, ^#^*P* < 0.05.

We further implemented rescue assays to investigate whether LINC00659 represses HCC cell proliferation and migration by regulating SLC10A1. Before this, we silenced SLC10A1 expression in HepG2 and HuH-7 cells (Figure S3(d)). Colony formation assay results demonstrated that LINC00659 overexpression inhibited the proliferation of HepG2 and HuH-7 cells, while knockdown of SLC10A1 partially reversed this effect (Figure 5(a)). Meanwhile, transwell assay showed that the suppressed cell migration caused by enforced expression of LINC00659 was partly offset after SLC10A1 (Figure 5(b)). Similarly, seahorse assay showed that the decreased ECAR and increased OCR in HepG2 and HuH-7 cells triggered by overexpressed LINC00659 could be partially restored by co-transfection of sh-SLC10A1-1 (Figures 5(c) and 5(d)). These results suggested LINC00659 suppressed cell proliferation, migration, and aerobic glycolysis in HCC and by regulating SLC10A1.

## 4. Discussion

HCC is the leading cause of cancer-related death and known as the major form of liver cancer worldwide. Therefore, the investigations into pathobiology of HCC are urgently needed to help patients' survival. Studies have shown that HCC causes reconstructions of glucose metabolism, from respiration to aerobic glycolysis, known as the phenomenon called “Warburg Effect”, supporting rapid cancer cell growth, survival, and invasion [33]. Recent reports have focused much on the effect of lncRNAs on Warburg Effect. Sun et al. have elucidated that downregulating lncRNA CASC7 inhibits tumor proliferation by reducing glycolysis through miR-143-3p/HK2 axis [34]. There is also report stating that lncRNA NEAT1 depletion inhibits aerobic glycolysis of prostate cancer cells accompanied by the reduction of lactate levels in the medium [35]. Li et al. have proved that lncRNA CYB561-5 promotes aerobic glycolysis and tumorigenesis by interacting with basigin in non-small cell lung cancer [36]. Our findings were consistent with the previous work, elucidating the role of LINC00659 in the aerobic glycolysis of HCC. We discovered that LINC00659 was lowly expressed in HCC tissues and cells. Overexpression of LINC00659 inhibited HCC cell proliferation, migration, and aerobic glycolysis. For all we know, LINC00659 has been reported to function as oncogenes in a variety of cancers, such as gastric cancer and colorectal cancer [37, 38]. However, our study for the first time demonstrated the tumor-suppressor potential of LINC00659 in HCC.

It has been proved that SLC10A1 inhibits HCC cell glycolysis by glucose utilization, lactate production, and extracellular acidification ratio [25]. Nguyen et al. found that SLC10A1 expression is an independent predictor of survival outcome and recurrence-free survival, implying that SLC10A1 is a potential biomarker for early diagnosis and prognosis of HCC in the era of personalized medicine [26]. Thus we raised the presumption that the inner mechanism of LINC00659 in HCC progression and aerobic glycolysis via regulation of SLC10A1. In our work, the expression of SLC10A1 was promoted in HCC cells transfected with pcDNA3.1-LINC00659, detected by qRT-PCR and WB, suggesting that LINC00659 positively regulated SLC10A1 expression in HCC cells. We also confirmed the phenomenon that SLC10A1 had low expression in HCC cells, which was consistent with previous work [39, 40].

To further explore the interaction between LINC00659 and SLC10A1, we applied starBase website to predict FUS could bind both to LINC00659 and SLC10A1, which was further confirmed by RIP and RNA pull-down results. Moreover, we proved that after FUS silence, the mRNA and protein levels of SLC10A1 were correspondingly reduced. Emerging evidence have unveiled that FUS regulates mRNA expression via interacting with mRNA 3′untranslated regions (UTRs) to stabilize mRNA [41]. Consistently, our research further found that FUS stabilized SLC10A1 expression. In addition, our study demonstrated that LINC00659 stabilized SLC10A1 mRNA and promoted its expression by recruiting FUS, which was similar with a variety of previous works. For example, lncRNA DUXAP8 is found to facilitate multiple malignant phenotypes and resistance to PARP inhibitor in HCC via binding with FUS [42]. Also, LINC01419 facilitates HCC growth and metastasis through targeting enhancer of zeste homolog 2 (EZH2)-regulated reversion-inducing-cysteine-rich protein with kazal motifs (RECK) through interacting with FUS [28]. However, in the future, there are still some long-term improvements to be taken in our study, for example, it is shown that SLC10A1 interacts with other proteins, such as NR1H4, ABCB11, and CYP7A, but we have not validated these candidates to rule out whether these proteins are involved in SLC10A1 modulation by LINC00659. Exploring more RNA-binding proteins (RBPs) involved in SLC10A1 modulation by LINC00659 is the direction of our next phase of work. In conclusion, our findings demonstrated that LINC00659 positively regulated SLC10A1 expression in HCC cells. Further, we found that LINC00659 stabilized SLC10A1 mRNA and promoted its expression by recruiting FUS. These findings elucidated that LINC00659 affected the aerobic glycolysis of HCC cells through FUS/SLC10A1 axis. Our results delineate the clinical significance of LINC00659 in HCC and the regulatory mechanisms involved in HCC progression, providing a novel prognostic indicator and promising therapeutic target.

## Data Availability

The datasets used and/or analyzed during the current study available from the corresponding author on reasonable request.

## Supplementary Materials

Supplementary Materials Bioinformation analysis results and related gene and protein detection results. Figure S1. (a) The expression of SLC10A1 in HCC tissues analyzed by GEPIA. (b) The overall survival and disease-free survival of HCC patients in low/high SLC10A1 group. (c) SLC10A1 expression in HCC tissue was stained using immunohistochemistry. (d) The overexpression efficiency of pcDNA-SLC10A1 in HepG2 and HuH-7 cells. ∗*P* < 0.05, ∗∗*P* < 0.01. Figure S2. (a) The expression of LINC00659 in HCC tissues analyzed by GEPIA. (b) The correlation between LINC00659 and SLC10A1 expressions in HCC samples. (c) The overexpression efficiency of pcDNA-LINC00659 in HepG2 and HuH-7 cells. ∗*P* < 0.05, ∗∗*P* < 0.01. Figure S3. (a) The RBP filtered by CLIP-DaTa ≥ 2 and CLIP-DaTa ≥ 3 on starBase. (b) FUS expression in HCC tissue was stained using immunohistochemistry. (c) The interference efficiency of sh-FUS in HepG2 and HuH-7 cells. (d) The interference efficiency of sh-SLC10A1 in HepG2 and HuH-7 cells. ∗∗*P* < 0.01.
