# Supplementary material for: LINC00659 Inhibits Hepatocellular Carcinoma Malignant Progression by Blocking Aerobic Glycolysis through FUS Recruitment and SLC10A1 Modulation
Source: Anal Cell Pathol (Amst). 2023 May 17;2023:5852963. doi: 10.1155/2023/5852963 (PMC10208759; doi:10.1155/2023/5852963)
Supplement: Supplementary Materials — Bioinformation analysis results and related gene and protein detection results. Figure S1. (a) The expression of SLC10A1 in HCC tissues analyzed by GEPIA. (b) The overall survival and disease-free survival of HCC patients in low/high SLC10A1 group. (c) SLC10A1 expression in HCC tissue was stained using immunohistochemistry. (d) The overexpression efficiency of pcDNA-SLC10A1 in HepG2 and HuH-7 cells. ∗P < 0.05, ∗∗P < 0.01. Figure S2. (a) The expression of LINC00659 in HCC tissues analyzed by GEPIA. (b) The correlation between LINC00659 and SLC10A1 expressions in HCC samples. (c) The overexpression efficiency of pcDNA-LINC00659 in HepG2 and HuH-7 cells. ∗P < 0.05, ∗∗P < 0.01. Figure S3. (a) The RBP filtered by CLIP-DaTa ≥ 2 and CLIP-DaTa ≥ 3 on starBase. (b) FUS expression in HCC tissue was stained using immunohistochemistry. (c) The interference efficiency of sh-FUS in HepG2 and HuH-7 cells. (d) The interference efficiency of sh-SLC10A1 in HepG2 and HuH-7 cells. ∗∗P < 0.01. [file 5852963.f1.docx]

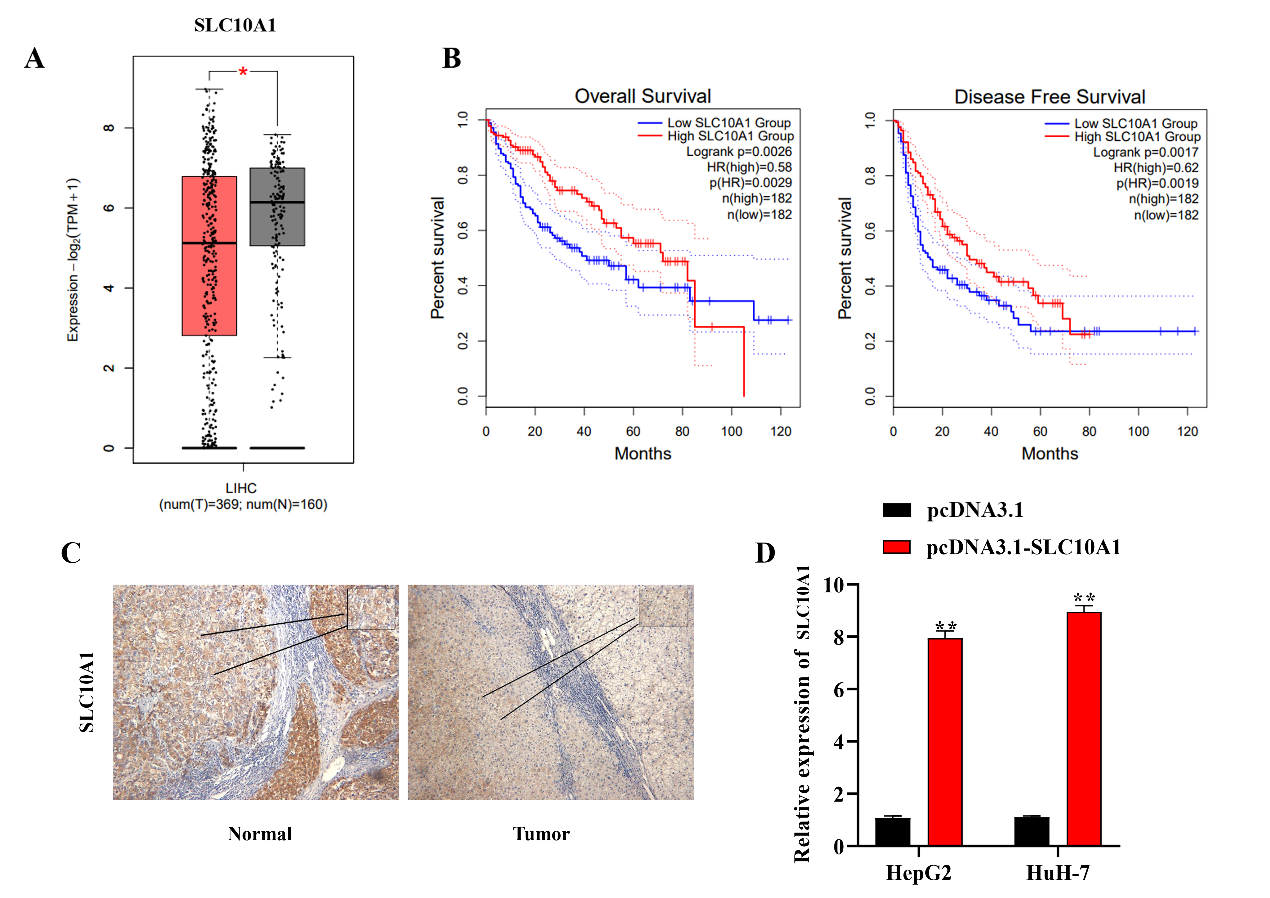


**Figure S1.** (**A**) The expression of SLC10A1 in HCC tissues analyzed by GEPIA. (**B**) The overall survival and disease free survival of HCC patients in low/high SLC10A1 group. (**C**) SLC10A1 expression in HCC tissue was stained using immunohistochemistry. (**D**) The overexpression efficiency of pcDNA-SLC10A1 in HepG2 and HuH-7 cells. ^*^P<0.05, ^**^P<0.01.


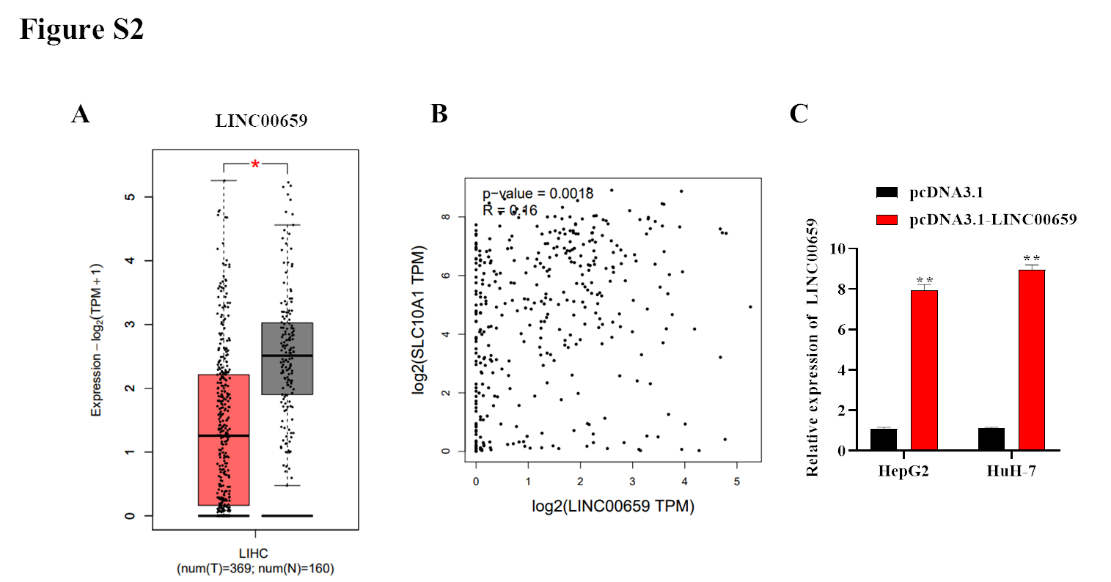


**Figure S2.** (**A**) The expression of LINC00659 in HCC tissues analyzed by GEPIA. (**B**) The correlation between LINC00659 and SLC10A1 expressions in HCC samples. (**C**) The overexpression efficiency of pcDNA-LINC00659 in HepG2 and HuH-7 cells. ^*^P<0.05, ^**^P<0.01.


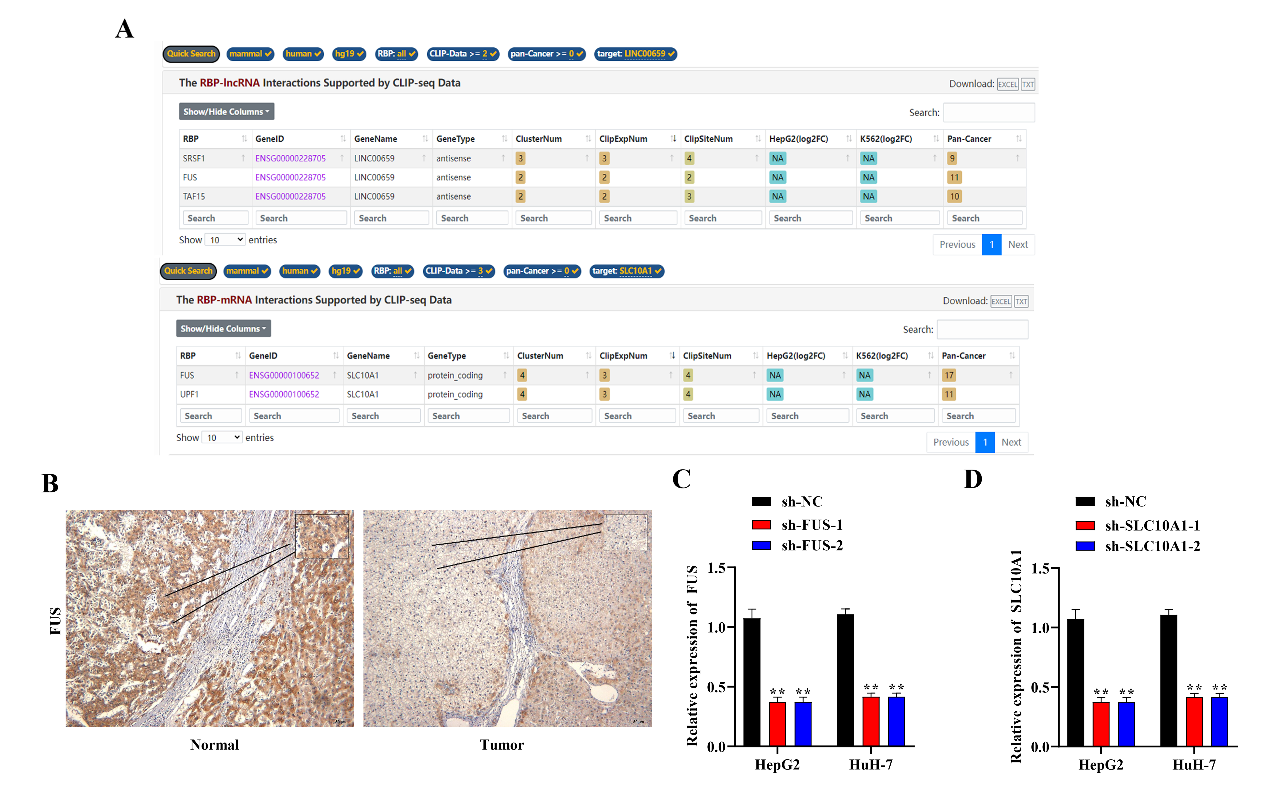


**Figure S3.** (**A**) The RBP filtered by CLIP-DaTa≥2 and CLIP-DaTa≥3 on StarBase. (**B**) FUS expression in HCC tissue was stained using immunohistochemistry. (**C**) The interference efficiency of sh-FUS in HepG2 and HuH-7 cells. (**D**) The interference efficiency of sh-SLC10A1 in HepG2 and HuH-7 cells. ^**^P<0.01.
